# The importance of choice disability and structural intervention in the HIV epidemic in Sub-Saharan Africa

**DOI:** 10.1371/journal.pone.0175297

**Published:** 2017-04-11

**Authors:** Rebecca de Boer, Jeffrey Musgrave, Neil Andersson, Frithjof Lutscher

**Affiliations:** 1 Department of Mathematics and Statistics, University of Ottawa, Ottawa, Ontario, Canada; 2 Department of Family Medicine, McGill University, Montreal, Quebec, Canada; 3 Department of Biology, University of Ottawa, Ottawa, Ontario, Canada; University of Toronto, CANADA

## Abstract

**Background:**

Despite massive investment in HIV control programs, HIV incidence rates in countries with generalized epidemics have not fallen for most of the last decade. It appears that those at risk are not adopting effective prevention strategies. Those who are unable to implement their prevention preferences are referred to as choice disabled. We examined how and to what extent structural intervention measures that support choice-disabled individuals can reduce HIV transmission and prevalence.

**Methods:**

A mathematical model was developed to describe HIV transmission among and between choice-disabled and choice-enabled individuals. Data were available from field trials identifying factors and effects of choice disability. The model was used to estimate the potential impact of an intervention strategy in which choice-disabled individuals are enabled to make prevention choices. Several scenarios were considered and compared: supporting only one or both genders; supporting only HIV– individuals or also HIV+ choice-disabled individuals.

**Results:**

Substantial declines in HIV incidence and prevalence are observed when supportive interventions are included in the model. The magnitude of these declines depends on the scope of the intervention program. The largest positive effect occurs when the support program is offered regardless of HIV status.

**Conclusions:**

Addressing the effects of choice disability in any HIV intervention program could be crucial to the program’s success. Structural intervention programs to support choice-disabled individuals in implementing prevention strategies greatly reduce HIV incidence and prevalence in mathematical models.

## Introduction

The successes in countering the generalised HIV epidemics in countries with the highest HIV rates like Botswana and Swaziland seem to have bottomed out [1]. For most of the last decade, incidence rates in these countries have remained in the region of 1.3-1.5% per year. In these hyper-endemic countries, young women carry a disproportionate risk of HIV. Incidence rates rise rapidly through the teenage years, and around one third of women are infected by their mid-20s [2].

Efforts to interpret these trends—excessive risk for young women and bottoming out of incidence despite massive investment in conventional HIV control programmes—suggest that those at risk are not adopting effective prevention strategies. The term *choice disabled* describes the inability of some people to implement prevention measures that might otherwise be very logical [2]. Severely inebriated people, victims of sexual violence, survivors of sexual violence with damaged self-esteem, and people who fear violent consequences are all less able to insist on their prevention preferences [3]. In the context of implementing HIV prevention options, they are choice disabled.

How much choice disability matters depends on the prevalence of choice disability and the relevance of HIV preventive strategies in this group. The iconic view of HIV prevention however, implies that people have the choice to abstain, chosen rather than forced sexual debuts, options to choose the number of partners, and the ability to insist on use of condoms. These strategies are much less relevant to the choice disabled than to their choice-enabled peers.

Observing the failure of conventional prevention strategies like promoting condoms to halt the epidemic, recent years have seen a surge of interest and investment in combination and structural interventions that change the objective position of those who are most at risk [4, 5]. Many of these focus on young women [6–10]. At least one country, Botswana, is currently undertaking a national trial of structural interventions to reduce HIV risk among young women [4].

In the current study, a mathematical model is employed to describe heterosexual HIV transmission in a generalized HIV epidemic. The model is based on a highly successful class of disease transmission models that have been applied to a wide variety of infectious diseases, including many aspects of HIV. Recently such models have been used to consider the effects of condom use in HIV control [11], treatment as prevention programs and drug resistance [12], and the potential impact of male circumcision on HIV transmission [13]. A closely related model forms the core of the UNAIDS Spectrum package for estimating HIV prevalence and incidence trends [14, 15].

## Methods

We use a mathematical model to investigate the potential results of a structural intervention aimed at individuals who are not in a position to implement their prevention choices, i.e. those who are choice disabled.

**Fig 1:**
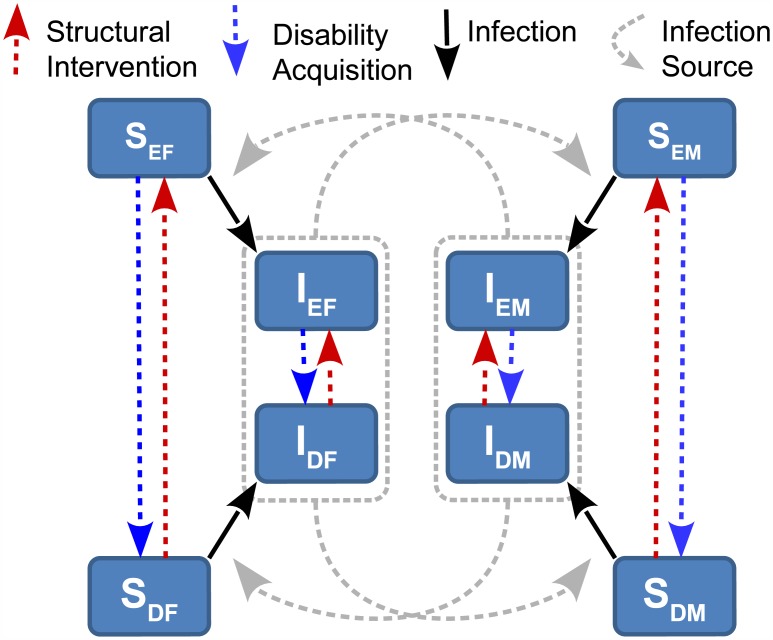
Flow chart of the infection and intervention processes in the model. The eight subgroups are represented by boxes and letters according to disease status (susceptible (S) and infectious (I)) and further distinguished with subscripts according to choice status (enabled (E) and disabled (D)) and sex (male (M) and female (F)).

The model focuses on heterosexual transmission of HIV between sexually active 15 to 29 year olds in Africa. The model identifies each individual in three categories: sex (male/female), disease status (susceptible/infectious), and choice status (enabled/disabled), producing eight subgroups. Mathematically, the model consists of a system of differential equations that track the number of individuals in each subgroup over time as individuals enter or leave the population, susceptible individuals become infectious, and individuals become choice-disabled or regain choice-enabled status through structural interventions. Each of these processes is described below and the model is summarized by Fig 1. The equations for the model are discussed in detail in the mathematical details section below.

**Demographic Processes**: Individuals enter one of the eight subgroups when they become sexually active and leave the population when they turn 30 years old or cease being sexually active.

**Disease Transmission**: A susceptible woman can become infectious through sexual activity with an infectious man, choice enabled or disabled, and similarly, susceptible men can become infectious through sexual activity with infectious women of either choice class.

As choice status is often associated with social or economic class we do not expect that individuals choose sexual partners independent of choice status. Instead, individuals of different choice classes participate in overlapping communities or social groups, such as those based on social and educational background. To describe the resulting mixing of individuals mathematically, we define *activity patterns* that describe the number of sexual partners that an individual has from within each of these communities. We consider several possible activity patterns.

The probability of disease transmission in the event of sexual contact between an infectious and susceptible individual depends on gender and choice status. Risk can be greatly reduced through condom use, or increased by engaging in dry or anal sex [16–18]. Those who are choice disabled are less likely able to chose safer sex practices. Similarly, the total number of sexual contacts may depend on choice status as those who are choice disabled may not be able to chose their number of sexual contacts as they would wish.

**Acquiring Choice Disability**: While some individuals are already choice disabled at the age of 15 when they enter the sexually active population in our model, others will acquire choice disability sometime between the ages of 15 and 29. In this model, a fraction of the choice enabled population will acquire choice disability every year.

**Structural Intervention**: The structural interventions of the Botswana INSTRUCT program aim to reduce the impact of choice disability by putting choice-disabled individuals in a position where they share the benefits of the choice-enabled.

We implement such interventions in the model by moving a fraction of individuals from the choice-disabled to the choice-enabled group every year.

### Mathematical details

The equations track how the number of individuals in each of the eight subgroups changes over time. These expressions relate the instantaneous rate of change of the size of each subgroup on the left hand side to the different processes producing this change. For (*S*_*EF*_), the number of enabled susceptible women, the equation reads

$$\begin{array}{c} \frac{d}{dt}S_{EF}=e_{SEF}N_{0}-l_{SEF}S_{EF}-c_{EF}S_{EF}Pr\left(S_{EF}\to I_{EF}\right)+s_{SF}S_{DF}. \end{array}$$ (1)

In this equation, the first term describes the number of individuals entering the sexually active group as the total number of individuals entering (*N*_0_) multiplied by the fraction (*e*_*SEF*_) of individuals who enter the group *S*_*EF*_. The second term describes the number of individuals leaving as a leaving rate (*l*_*SEF*_) multiplied by the number of *S*_*EF*_. The third term is the infection term that reduces the number of *S*_*EF*_ by the contact rate (*c*_*EF*_) multiplied by the total number of *S*_*EF*_ and the probability that a single contact leads to infection (*Pr*(*S*_*EF*_ → *I*_*EF*_). The last term accounts for *S*_*DF*_ who become enabled by the rate of support (*s*_*SF*_) that they receive.

The other seven equations are constructed in the same fashion, taking into account that individuals who move between subgroups must be properly accounted for. In the equations for infectious subgroups, the infection process appears with a + sign as individuals are entering this class. On the other hand, the support process appears with a − sign in the choice disabled subgroups as individuals are leaving this class due to supportive interventions. The remaining equations are

$$\begin{array}{c} \frac{d}{dt}S_{EM}=e_{SEM}N_{0}-l_{SEM}S_{EM}-c_{EM}S_{EM}Pr\left(S_{EM}\to I_{EM}\right)+s_{SM}S_{DM}\\ \frac{d}{dt}S_{DF}=e_{SDF}N_{0}-l_{SDF}S_{DF}-c_{DF}S_{DF}Pr\left(S_{DF}\to I_{DF}\right)-s_{SF}S_{DF}\\ \frac{d}{dt}S_{DM}=e_{SDM}N_{0}-l_{SDM}S_{DM}-c_{DM}S_{DM}Pr\left(S_{DM}\to I_{DM}\right)-s_{SM}S_{DM}\\ \frac{d}{dt}I_{EF}=e_{IEF}N_{0}-l_{IEF}I_{EF}+c_{EF}S_{EF}Pr\left(S_{EF}\to I_{EF}\right)+s_{IF}I_{DF}\\ \frac{d}{dt}I_{EM}=e_{IEM}N_{0}-l_{IEM}I_{EM}+c_{EM}S_{EM}Pr\left(S_{EM}\to I_{EM}\right)+s_{IM}I_{DM}\\ \frac{d}{dt}I_{DF}=e_{IDF}N_{0}-l_{IDF}I_{DF}+c_{DF}S_{DF}Pr\left(S_{DF}\to I_{DF}\right)-s_{IF}I_{DF}\\ \frac{d}{dt}I_{DM}=e_{IDM}N_{0}-l_{IDM}I_{DM}+c_{DM}S_{DM}Pr\left(S_{DM}\to I_{DM}\right)-s_{IM}I_{DM}. \end{array}$$ (2)

The probability of infection depends on the activity pattern of the individual, the probability of encountering an infectious partner, the type of sexual activity, and the transmission probability per sex act. For a member of the group *S*_*EF*_, this probability is

$$\begin{array}{l} Pr\left(S_{EF}\to I_{EF}\right)=\\ a_{EF}\left(f_{EF,EM}^{\left(1\right)}\frac{I_{EM}}{I_{EM}+S_{EM}}t_{EM\to EF}+f_{EF,DM}^{\left(1\right)}\frac{I_{DM}}{I_{DM}+S_{DM}}t_{DM\to EF}\right)\\ +\left(1-a_{EF}\right)\left(f_{EF,EM}^{\left(2\right)}\frac{I_{EM}}{I_{EM}+S_{EM}}t_{EM\to EF}+f_{EF,DM}^{\left(2\right)}\frac{I_{DM}}{I_{DM}+S_{DM}}t_{DM\to EF}\right). \end{array}$$ (3)

To interpret this expression, notice that a member of *S*_*EF*_ chooses the first activity pattern with probability *a*_*EF*_ and the second activity pattern with probability (1 − *a*_*EF*_). In the first activity pattern, a fraction $f_{EF,EM}^{\left(1\right)}$ of contacts is with an enabled male, and a fraction $f_{EF,DM}^{\left(1\right)}$ is with a disabled male. The probability that a contact with an enabled male, once it occurs, is with an infectious partner is the same as the fraction of enabled infectious males among all enabled males. Please note that this does not mean that the contact itself occurs with the same probability as the proportion within the population. The probability of contact is determined by the activity patterns. Finally, the probability of disease transmission in such a contact is *t*_*EM*→*EF*_.

The fraction of contacts of an enabled female with an enabled male in the first activity pattern according to proportionate mixing [19] is the number of contacts made by enabled males in the first activity pattern divided by the total number of contacts made in that activity. That is,

$$\begin{array}{l} f_{EF,EM}^{\left(1\right)}=\\ \frac{c_{EM}a_{EM}\left(S_{EM}+I_{EM}\right)}{\left(\begin{array}{ll} c_{EM}a_{EM}\left(S_{EM}+I_{EM}\right) & +c_{EF}a_{EF}\left(S_{EF}+I_{EF}\right)\\ & +c_{DM}a_{DM}\left(S_{DM}+I_{DM}\right)+c_{DF}a_{DF}\left(S_{DF}+I_{DF}\right) \end{array}\right)} \end{array}$$ (4)

### Parameter estimation

To simulate the outcome of the model, we need to specify parameter values for all of these processes. Some of these values are reported in the literature, for others, only ranges are available.

Of the approximately 50,000 fifteen year olds in Botswana, roughly 80% have debuted sexually, so that about 40,000 individuals enter the population in our model each year. We assume a 50:50 sex ratio. According to a recent study from Botswana [2], half of the girls in the incoming population are choice disabled, and 5.6% of the choice-disabled girls are HIV positive. Furthermore, all of the choice enabled girls are HIV negative. None of the 15 year old boys are HIV positive, but 12% are choice disabled (all data from the same study).

As the model considers a 15-year age range, about 1/15 = 6.67% of the population leaves each year.

A typical rate for sexual contacts in the literature is approximately 80 per year. For example, Gray et al. [16] found that monogamous, heterosexual HIV-discordant couples in Uganda have intercourse 8-9 times per month. There are no studies on how this number changes for choice disabled individuals, but it could be considerably higher when transactional sex is an economic necessity. We will compare two scenarios, one in which all individuals make 80 contacts per year and one in which choice disabled individuals make three times as many contacts.

Boily et al. [20] conducted a systematic review of observational studies to estimate transmission probabilities of HIV per vaginal sex act. They found the probability of HIV transmission from male to female to be 0.0038 and from female to male to be 0.0030 in the absence of condom use. With condom use, these numbers decrease 20 fold [17]. Typically, choice-enabled individuals seem to use condoms 65% of the time so that the total transmission probability from enabled men to women is 0.0015 and from women to men is 0.0011.

Higher risk sexual behaviour increases transmission probabilities. For example, dry sex is very common in South Africa, especially in younger individuals with low education [18], and it increases the transmission by a factor of 5. Anal sex is riskier than vaginal sex and increases transmission probability by factors of 1.5 for the insertive partner and 5 for the receptive partner [17]. Probability of transmission is computed based on the choice class of the individuals involved by making assumptions about the frequency of condom use and high risk sexual activity.

For sexual contacts between choice enabled women and choice disabled men, we assume condom use 65% of the time, but half of the non-condom contacts will be high-risk activities such as anal or dry sex. Then the transmission probabilities are 0.0014 from women to the men and 0.0041 from men to the women.

For contacts between choice enabled men and choice disabled women, we assume that all of the non-condom sexual activity is high risk. This results in a transmission probability of 0.0068 from men to women and 0.0017 from women to men.

When both partners are choice disabled, we assume that condoms are used half as often as in the previous cases, and that 35% of sex acts are high risk. Then the transmission probabilities are 0.0079 from men to women and 0.0026 from women to men.

To be consistent with our choice of model parameters, initial conditions are also taken from data collected in Botswana [2], and the choice disability acquisition rates are calibrated so that neither the overall population nor any of the choice class populations change size during model simulations when there is no structural intervention.

We now define the activity patterns that model how individuals in the various choice classes interact. We consider a scenario where people interact within two overlapping communities: a high-status community dominated by the choice enabled and a low-status community dominated by the choice disabled. We describe two different scenarios.

In the first scenario individuals mostly interact with those of similar backgrounds. Choice enabled individuals make 80% of their sexual contacts within the high status community and the remaining 20% in the low status community. Meanwhile, choice disabled individuals show the reverse pattern making only 20% of their sexual contacts in the high-status community and 80% of their contacts low-status community.

In the second scenario, transactional sex is included by considering an activity pattern where choice-enabled individuals more often seek sexual encounters with those who are choice disabled. The choice enabled make only 60% of their contacts within the high-status community and the remaining 40% within the low-status community.

The model is used to consider a structural intervention that moves a constant fraction of the choice-disabled population into the choice-enabled population every year. In the model, this fraction can range between 0 and 13%, depending on the strength of the intervention program. The corresponding rate parameter in the differential equation model ranges from 0 to 2 per year since exp(−2) ≈ 13%. Interpreted differently, these figures imply that individuals are on average choice enabled for at least 6 months before they benefit from intervention.

All parameters, together with their meaning and base numerical value as used in model simulations are listed in Table 1.

**Table 1:** Description and baseline values of parameters used in the model. Indices refer to disease status as susceptible (*S*) or infectious (*I*); to choice class as enabled (*E*) or disabled (*D*); and to sex as female (*F*) or male (*M*).

Parameter	Description	Value
*N*_0_	Number of new sexually active individuals	40 000 per year
*e*_*SEF*_	Fraction entering *S*_*EF*_	25%
*e*_*SEM*_	Fraction entering *S*_*EM*_	44%
*e*_*SDF*_	Fraction entering *S*_*DF*_	22.2%
*e*_*SDM*_	Fraction entering *S*_*DM*_	6%
*e*_*IEF*_	Fraction entering *I*_*EF*_	0%
*e*_*IEM*_	Fraction entering *I*_*EM*_	0%
*e*_*IDF*_	Fraction entering *I*_*DF*_	2.8%
*e*_*IDM*_	Fraction entering *I*_*DM*_	0%
*l*_*SEF*_	Leaving rate for *S*_*EF*_	0.0667
*l*_*SEM*_	Leaving rate for *S*_*EM*_	0.0667
*l*_*SDF*_	Leaving rate for *S*_*DF*_	0.0667
*l*_*SDM*_	Leaving rate for *S*_*DM*_	0.0667
*l*_*IEF*_	Leaving rate for *I*_*EF*_	0.0667
*l*_*IEM*_	Leaving rate for *I*_*EM*_	0.0667
*l*_*IDF*_	Leaving rate for *I*_*DF*_	0.0667
*l*_*IDM*_	Leaving rate for *I*_*DM*_	0.0667
*c*_*EF*_, *c*_*EM*_	Contact rates for enabled individuals	80 per year
*c*_*DF*_, *c*_*DM*_	Contact rates for disabled individuals	80–240 per year
*t*_*EM*→*EF*_	Transmission probability from *I*_*EM*_ to *S*_*EF*_	0.0015
*t*_*EF*→*EM*_	Transmission probability from *I*_*EF*_ to *S*_*EM*_	0.0011
*t*_*DM*→*EF*_	Transmission probability from *I*_*DM*_ to *S*_*EF*_	0.0041
*t*_*EF*→*DM*_	Transmission probability from *I*_*EF*_ to *S*_*DM*_	0.0014
*t*_*EM*→*DF*_	Transmission probability from *I*_*EM*_ to *S*_*DF*_	0.0068
*t*_*DF*→*EM*_	Transmission probability from *I*_*DF*_ to *S*_*EM*_	0.0017
*t*_*DM*→*DF*_	Transmission probability from *I*_*DM*_ to *S*_*DF*_	0.0079
*t*_*DF*→*DM*_	Transmission probability from *I*_*DF*_ to *S*_*DM*_	0.0026
*a*_*EF*_, *a*_*EM*_	Activity patterns for enabled individuals	0.6-0.8
*a*_*DF*_, *a*_*DM*_	Activity patterns for disabled individuals	0.2
*s*_*SF*_, *s*_*IF*_, *s*_*SM*_, *s*_*IM*_	Supportive intervention	0–2

### Procedure

The model is used to estimate HIV prevalence and annual incidence rate over a 15 year period. Standard numerical software is used to solve the differential equations (Matlab). Various scenarios involving contact rates, activity patterns, and level of intervention support are considered.

Since many of the parameter estimates are uncertain, in addition to considering the baseline parameter values described above, estimates were computed for a sample of possible parameter values. We considered that each of the disease transmission and intervention parameters could vary by 20% around its base value and chose a sample of 10 000 possible sets of parameter values using Latin Hypercube Sampling [21–23].

The sample also allowed for a thorough sensitivity analysis to be performed using partial rank correlation coefficients. These coefficients quantify the influence of each of the sampled parameters on the resulting HIV prevalence estimates while controlling for the influences of other co-varying parameters.

## Results

**Fig 2:**
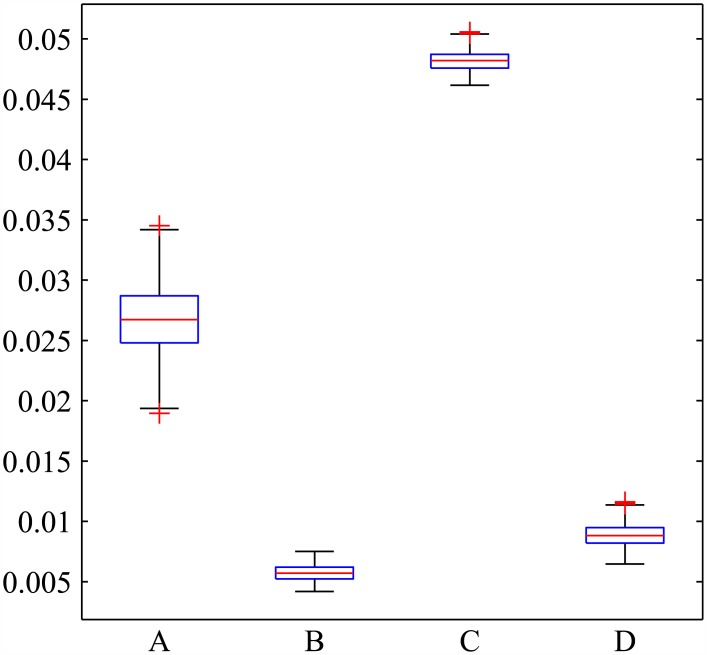
Annual incidence rate in several scenarios. The box and whisker plots show the median annual incidence rates for the sampled parameter values in several cases. In A and B, the number of contacts made by an individual is independent of choice class while in C and D choice disabled individuals make three times as many contacts. Panels A and C have no structural intervention while B and D implement the maximum supportive intervention for the choice disabled.

The model estimates of annual HIV incidence for the sampled parameter set is summarized in Fig 2. The box and whisker plots give the median, the first and third quartile, as well as the maximum and minimum values. When the number of sexual contacts per year is independent of choice class and no structural intervention is implemented, the annual HIV incidence rate has a median value of 2.67% (IQR 2.48% to 2.87%). When the maximum intervention is implemented this median drops to 0.57% (IQR 0.52% to 0.62%). The scenario where choice disabled individuals have more contacts results in higher estimated incidences: without intervention the median is 4.82% (IQR 4.76% to 4.87%), with maximum intervention the median drops to 0.88% (IQR 0.82% to 0.95%).

**Fig 3:**
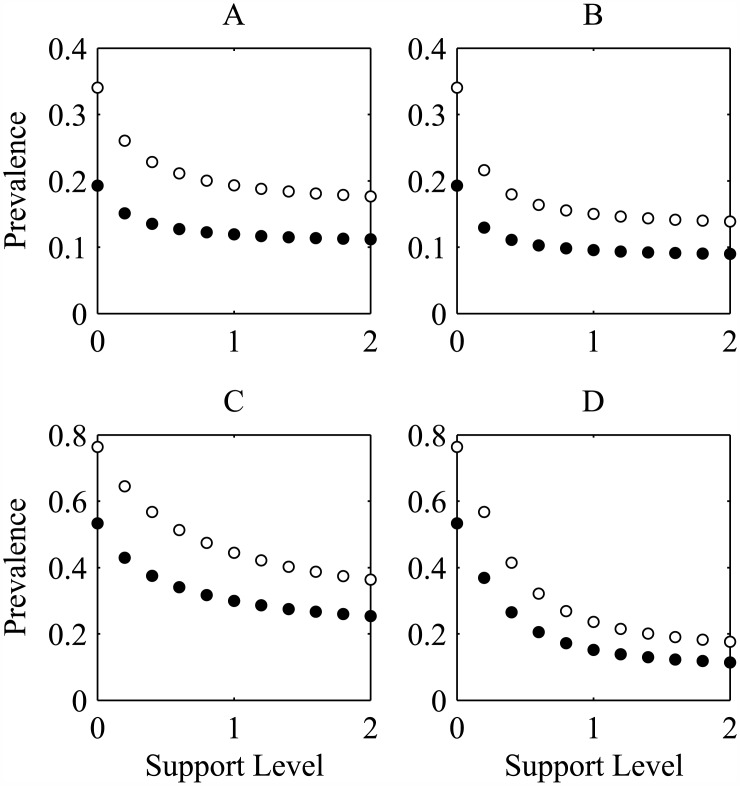
15 year prevalence. The four panels show HIV prevalence after a 15 year period for different support levels and contact scenarios for men (solid circles) and women (open circles). In A and B the number of contacts made by an individual is independant of choice class while in C and D choice disabled individuals make three times as many contacts. For A and C, only HIV-negative individuals receive support while in B and D everyone benefits from the support intervention.

The effects of various intervention implementations are shown in Fig 3. This figure displays HIV prevalence 15 years after implementation of interventions of various strength. Increasing the strength of the intervention results in a decrease in the HIV prevalence after 15 years. This decline is particularly pronounced when all choice disabled individuals are supported by the intervention.

When choice enabled and disabled individuals make the same number of yearly contacts, HIV prevalence in women (men) is estimated at 34.0% (19.3%) with no intervention. When an intervention targeting HIV-negative individuals is implemented, this estimate drops to 19.3% (11.9%) with an intervention where individuals remain choice disabled for 1 year on average. When both HIV positive and HIV negative individuals are included in the intervention, the estimated HIV prevalence is reduced further to 15.0% (9.6%)

When the overall contact rate is higher in choice disabled individuals then concentrating intervention on non-infections individuals only has a smaller effect than including all disease classes. In fact, even very strong support programs that target only the HIV-negative population lead to a higher prevalence after 15 years than moderate programs that include HIV-positive individuals.

**Fig 4:**
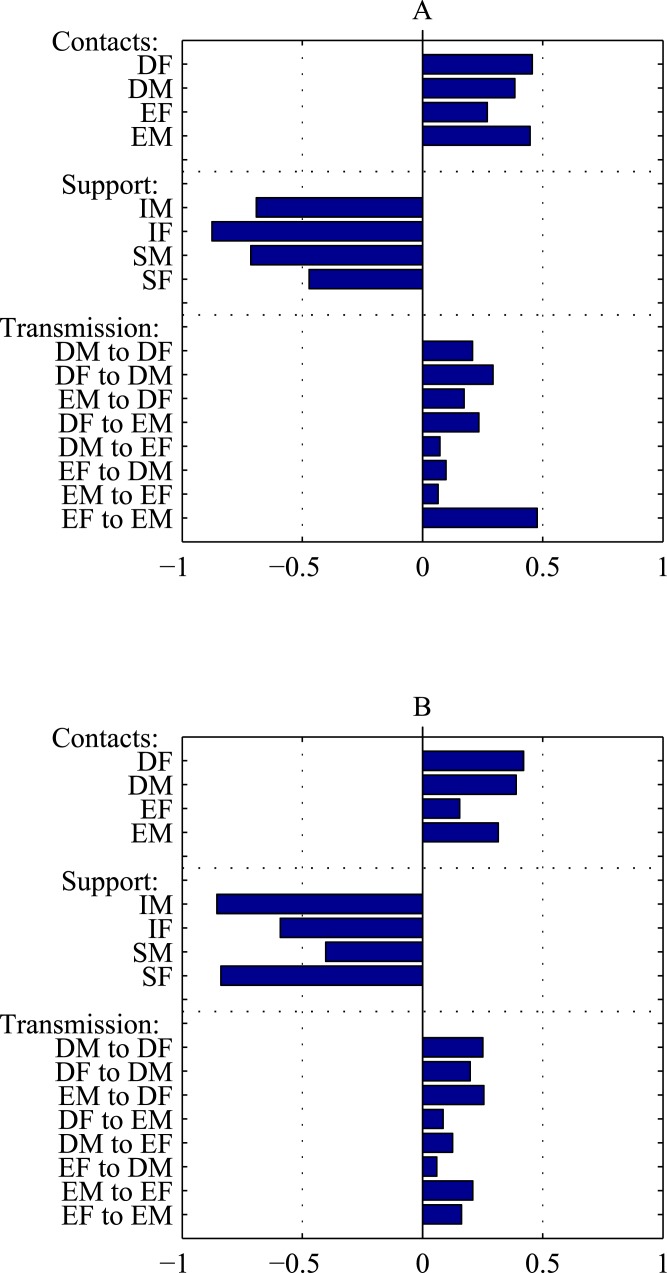
Sensitivity plot. The plot shows the partial rank correlation coefficients between model parameters and HIV prevalence in men (A) and women (B) after 15 years. The bottom eight bars refer to transmission probabilities between the various sex and choice classes. The middle four bars refer to the strength of the structural intervention. The top four bars refer to the number of contacts by each sex and choice class. In both plots, the intervention parameters are seen to be the most important as they show large negative rank correlations with HIV prevalence indicating that increasing the size of the intervention consistantly decreases HIV prevalence.

More insight about the influence that each mechanism and intervention has on HIV prevalence after a 15 year period is found in the sensitivity analysis summarized in Fig 4. The partial rank correlation coefficients between each of the parameters and the HIV prevalence for men and for women are displayed as bar graphs. A bar to the left indicates a negative correlation—increasing the parameter reduces HIV prevalence. A bar to the right indicates a positive correlation—increasing the parameter increases the annual incidence.

In general, the results of the sensitivity analysis are as expected. Increasing the number of contacts made by individuals in any of the choice and sex classes increases HIV prevalence. Similarly, increasing any of the transmission coefficients increases HIV prevalence. Increasing the intervention support level for any population results in a decrease in HIV prevalence. Due to their large correlations, the support levels are highlighted as most important for HIV prevalence while several of the contact rates are also important.

**Fig 5:**
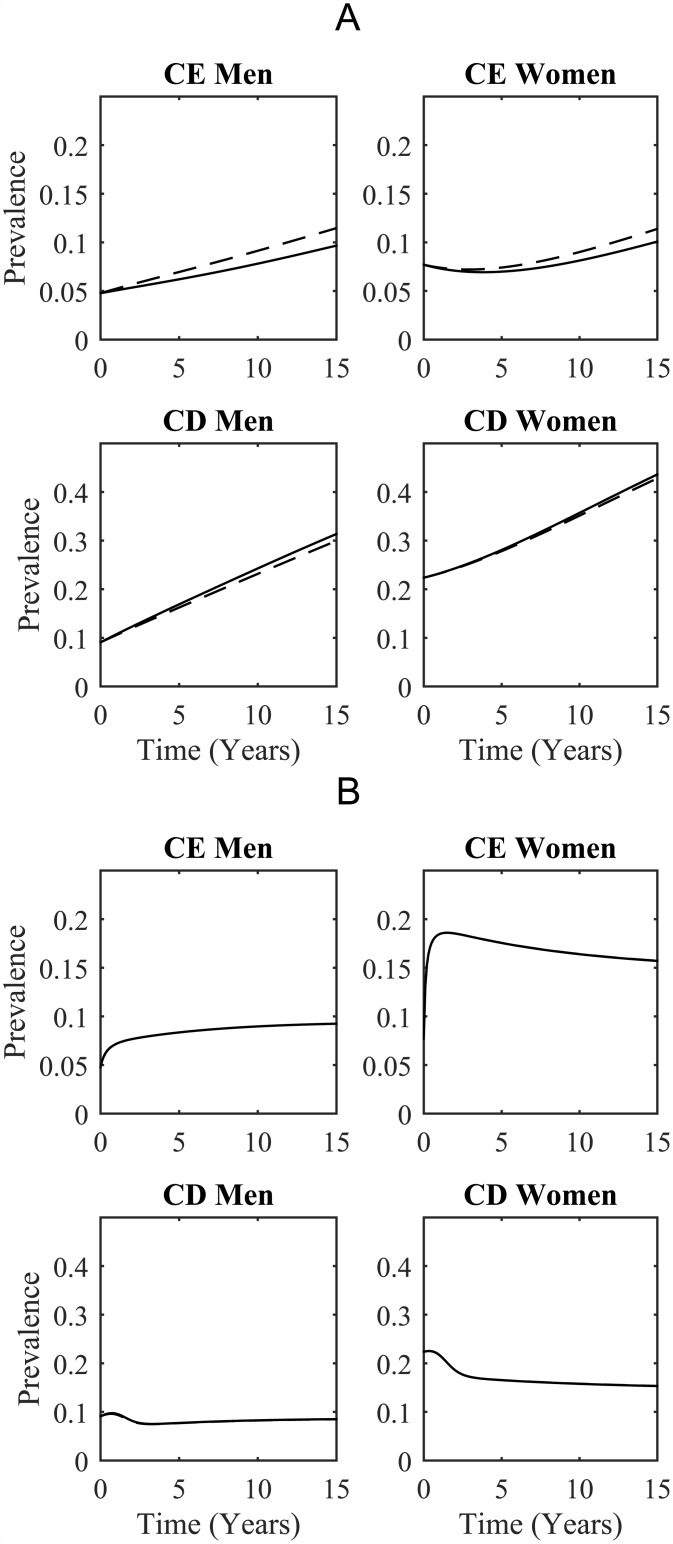
Comparison between base-line and alternative activity patterns. These plots show HIV prevalence in each of the choice and sex classes. In both cases, the number of contacts an individual makes is independent of their choice class. In A there is no structural intervention providing support to the choice disabled while in B the maximum intervention is used and individuals spend an average of only 6 months choice disabled. Please note that the scale for the prevalence in CE and CD compartments differ.

For a more detailed view of the changes in prevalence over the 15 year time period that we have been considering, and to see the effects of different activity patterns, see Fig 5. The four plots of panel A show the estimated prevalence in each choice and sex class over the next 15 years in the absence of a supportive intervention for the choice disabled. Overall HIV prevalence is seen to increase somewhat over this time period with higher prevalence in the choice-enabled population when a larger fraction of their contacts are made within the community dominated by the choice disabled.

The four plots of panel B show the estimated prevalence in each choice and sex class when an intervention providing support for the choice disabled is implemented. HIV prevalence in the choice-enabled classes is initially seen to increase rapidly as choice disabled people with high HIV prevalence are moved into the choice-enabled classes. Meanwhile, HIV prevalence in the choice-disabled classes drops. Very little difference is seen between the two activity patterns in this case.

## Discussion

The model in the current study is novel in that distinguishes between the choice disabled and the choice enabled, and it includes decreased ability of the choice disabled to make decisions to lower their risk of HIV. The model allows testing potential impacts and estimating effects of structural intervention programs for the choice disabled on HIV incidence and prevalence.

This mathematical model is the first to include choice disability in a disease transmission model. It is a high-level model to show proof of concept, independent of the particular causes and effects of choice disability and disregarding different levels of choice disability. The model illustrates how choice disability might explain the excessive risk among young women and how choice disability contributes to the limited impact of HIV prevention programs in sub-Saharan Africa.

The model results suggest a promising role for structural programs that ameliorate the effects of choice disability in HIV transmission prevention. It seems likely that differently implemented intervention programs will have different results. The modelled simulations suggest that the best results are achieved when those who are HIV positive benefit along with their HIV negative peers. The benefits of these types of programs may be particularly pronounced when the choice disabled have more sexual contacts than the choice enabled.

An examination of the sensitivity results for the model confirms that structural intervention programs could play an important role in HIV prevalence reduction, but that other factors, particularly the number of sexual contacts made by each group, should not be ignored in designing intervention programs.

Structural programs to support the choice disabled may also neutralize some of the HIV transmission effects of transactional sex. Including transactional sex into the model without an intervention program increased the overall HIV prevalence in the choice enabled population. However, when an intervention program is included the estimated prevalences were very similar with and without transactional sex.

There are several limitations to the current model. For simplicity, only two choice classes were considered: enabled and disabled. However, the literature shows that there are multiple levels of severity of choice disability that increase HIV risk farther. Two other factors that were also excluded to simplify the model are homosexual sexual activity and intravenous drug use. The extent to which these two factors contribute to transmission varies considerably with geographic region. Our simplification is consistent with a recent review study that showed that heterosexual transmission dominates in sub-Saharan Africa, whereas drug use and sex work contribute significantly in North Africa and the Middle East, and homosexual sexual activity is the greatest contributor in Latin America [24]. Finally, medical HIV treatment programs are not included although choice disability could also impact access to treatment. Any effects that HIV infection could have on choice disability or on sexual behaviour are also excluded.

To parameterize the model, data was pooled from different countries and demographics, but very few of these studies address choice disability directly. The model uses the Botswana population as illustrative and would need adjustment for other contexts.

Future work will be needed to deal with these complexities. Models can be developed that take choice disability into account along with other factors such as multiple transmission pathways, treatment programs, or more detailed effects of choice disability and HIV infection. Such models could provide a more nuanced picture of the causes of choice disability and its effects on HIV transmission.

The model was also developed for a high transmission/heterosexual behavior setting at the epicentre of the HIV pandemic. Therefore, careful consideration is needed to apply the model to lower transmission setting such as East Africa or to the concentrated epidemics of North America and Europe, where, in addition, homosexual transmission is often the driver of the epidemic [24].
